# Prevalence of, and Factors Associated with Intestinal Parasites in Multinational Expatriate Workers in Al Ain City, United Arab Emirates: An Occupational Cross-Sectional Study

**DOI:** 10.1007/s10903-019-00903-8

**Published:** 2019-06-27

**Authors:** Rami H. Al-Rifai, Tom Loney, Mohamud Sheek-Hussein, Sumaya Zoughbor, Suad Ajab, Marie Olanda, Zakeya Al-Rasbi

**Affiliations:** 1 Institute of Public Health, College of Medicine and Health Sciences, United Arab Emirates University, PO Box 17666, Al Ain, United Arab Emirates; 2 College of Medicine, Mohammed Bin Rashid University of Medicine and Health Sciences, PO Box 505055, Dubai, United Arab Emirates; 3 Microbiology and Immunology Department, College of Medicine and Health Sciences, United Arab Emirates University, PO Box 17666, Al Ain, United Arab Emirates

**Keywords:** Communicable diseases, Infectious disease transmission, One health, Parasitic intestinal diseases, Transients and migrants, United Arabia Emirates

## Abstract

To estimate the prevalence of, and identify factors associated with intestinal parasites (IPs) in expatriate workers in the United Arab Emirates (UAE). All expatriate workers (N = 115) in a conveniently selected workplace in the industrial district of Al Ain city were invited to participate in a cross-sectional study. Consenting workers completed an interviewer-led questionnaire and self-collected stool samples. Stool samples were microscopically and molecularly screened for the presence of IPs. Univariate and multivariate analyses were conducted. Overall, 102 (88.7%) workers participated in the survey and 84.3% provided stool samples. Over three-quarters (79.4%) of workers were living in labour accommodation, 76.0% were sharing a bedroom with ≥ 4 workers, 80.2% were sharing a toilet with > 5 other people. Fifteen species of IPs were identified. Microscopically, 17.4% of the screened stool samples were positive for at least one parasite. *Entamoeba* species was the most common (8.1%) followed by *Cryptosporidium* species (3.5%). Thirty-six (41.8%) of the tested stool samples were positive for at least one parasite by molecular testing. The most prevalent parasite was *Cryptosporidium* species (16.3%) followed by *Enterobius vermicularis* (14.0%) and *Ascaris lumbricoides* (5.8%). Overall, 47.8% of the tested expatriate workers were positive for at least one IP, microscopically or molecularly. Educational attainment was negatively associated with being positive for at least one IP. IPs were very common amongst expatriate workers in Al Ain city. Efficacious and cost-effective public health interventions are required to reduce the burden of, and prevent the onward transmission of IPs in the UAE.

## Introduction

Infectious intestinal parasites (IPs) including helminths and protozoans represent a subset of neglected diseases, particularly in developing and tropical countries [1]. From 1991 to 2008, 11% of the worldwide waterborne outbreaks were caused by parasites [2]. In 2010, the global burden of diseases caused by the major intestinal nematodes (round worms) were estimated at 450 million infected people with hookworm, approximately 800 million with *Ascaris lumbricoides*, 460 million with *Trichuris trichiura*, and 300 million with Schistosoma species [3]. In 2018, the World Health Organization (WHO) estimated that approximately 25% of the world’s population were infected with soil-transmitted helminths [4]. Five million years lived with a disability, were attributed to soil transmitted helminths, of which 65% were attributed to hookworms, 22% to *A. lumbricoides,* and the remaining 13% to *T. trichiura* [3, 5]. Exposure to IPs have several potential unfavourable health outcomes including, but not limited to, physical development [6, 7] and growth retardation [8, 9], depression, reduced intellectual capacity and memory [10], cancer [11, 12], and decreased female fecundity and fertility [13–15].

Cryptosporidium and giardia are the two most common waterborne parasitic infections leading to diarrhoea [16, 17]. Other waterborne protozoan IPs that cause human complications are; *Toxoplasma gondii*, *Entamoeba histolytica*, *Acanthamoeba* spp., *Cyclospora cayetanensis*, *Microsporidia*, *Isospora*, *Blastocystis hominis*, *Sarcocystis* spp., *Naegleria* spp. and *Balantidium coli* [2, 18]. Waterborne outbreaks of protozoan cryptosporidium infection have been documented all over the world in contaminated swimming pools, recreational and public water supplies [17], drinking water reservoirs, and contaminated food [19].

A wide range of socioeconomic, environmental, and hygienic factors contribute to the increased risk of contracting IPs. In recent decades, most countries in the Arabian Gulf peninsula have experienced substantial improvements in living standards for city inhabitants, mostly facilitated by the income from oil and gas reserves. The six Gulf Cooperation Council (GCC) countries (Bahrain, Oman, Kuwait, Qatar, Saudi Arabia, and United Arab Emirates; UAE) are categorised as high-income developing countries. The six GCC countries have different tropical disease control programs and almost all of them have been declared successful by the WHO [1, 18, 20]. However, rapid socio-economic developments and sustained economic stability have resulted in a mass influx of expatriate workers to these countries, mainly from less affluent and developed countries [21].

Patterns of parasitic infection vary within a population and are linked to countries of origin [22–25], host gender [7], and annual fluctuations in parasite transmission rates within a population pool [24, 26, 27]. The UAE is a rapidly developing country, composed of multinational populations with varied educational backgrounds, religious beliefs, eating and recreational habits and behaviours, and cultural practices [28]. The annual population growth for the UAE is approximately 3.3% [21], which places it sixth in the world rankings. Expatriates to the UAE mainly originate from developing countries in Africa, Asia, and South Asia [29, 30]; where parasitic infections are often endemic [31–33]. In the UAE, expatriates from India, Bangladesh, and Pakistan comprise over 80% of the country’s workforce [34]. In 1982, the Abu Dhabi Public Health Preventative Medicine Laboratory found that 34% of all expatriate food handlers working in the country had helminths and protozoan infections [35]. A retrospective analysis of stool samples collected from a convenient sample of healthy expatriate workers for routine residency visa health screening between January and December 2013 reported that 3.3% of 21,347 tested expatriate workers of different occupations were positive for IPs in the emirate of Sharjah (UAE); however, the stool samples were only analyzed microscopically without molecular identification [36].

Currently, there is a scarcity of data on the burden of IPs and its associated factors among expatriate workers in the UAE. This study aimed to estimate the prevalence of, and identify sociodemographic and health-related factors associated with IPs, in expatriate workers in the UAE.

## Materials and Methods

### Study Design and Subjects

A cross-sectional survey was conducted between April and July 2017 in an industrial district (Senaiya) of Al Ain city, in the emirate of Abu Dhabi, UAE. All multinational expatriate workers in a conveniently selected workplace were invited to participate in this study. Workers with abdominal surgery or who had recently taken antibiotic or anti-parasitic medication were excluded.

### Survey Instruments and Procedure

To our knowledge, there are no validated data collection instruments to assess potential factors that could be associated with exposure to IPs relevant for the contextual setting of our study in the UAE (i.e. multinational migrant workers with varying levels of education and different cultural practices). Hence, a structured questionnaire was developed by the study research team composed of a clinical microbiologist, epidemiologist, parasitologist, and a veterinarian. The questionnaire was specifically designed to collect information on socio-demographics, exposure to animals, living and accommodation conditions in the UAE, drinking and eating habits, travel history, self-rated stool type, and self-reported craving for sugar.

The questionnaire was repeatedly reviewed internally by the multilingual research team who were fluent in the spoken languages (Arabic, Hindi, Tagalog, and Urdu) of the target study population. However, to avoid any potential measurement bias and to ensure correct comprehension of the questions, the questionnaire was then piloted on a convenient sample of multinational workers who were employed in another workplace within the same industrial district. Minor modifications and re-wording were implemented following the piloting phase.

Native Arabic, Tagalog, and Urdu speaking interviewers and other research team members received 3 days of training to become conversant with the survey objectives and procedures. All face-to-face anonymous interviews collected information from the voluntary consenting workers. The quality of the data collection was standardized using repeated pretests until all research staff had achieved the required level of competency.

### Survey and Anthropometric Data

Survey questionnaires collected data on socio-demographic and living characteristics. Socio-demographics included age, education level, gender, marital status, ethnicity, place of residence in the home country, and length of stay in the UAE. Living and behavioral characteristics covered type of accommodation, number of people sharing the same bed room, number of people sharing the same toilet, frequency of eating unwashed food, most frequent source of food, using the same cutting board for vegetables and meat, and last time traveled abroad outside of the UAE. Health-related characteristics collected information on type of stool using the Bristol stool chart [37], frequency of craving for sugar, hemoglobin (Hb) concentration, and nutritional status. The Hb concentration was measured using a non-invasive device (Pronto Pulse Co-Oximeter, Masimo). Following the WHO guidelines [38], anemia was defined as Hb concentration < 12.0 g/dl in women and < 13.0 g/dl in men. To assess nutritional status, we computed the body mass index (BMI) by measuring the standing body height in centimeters (cm) and body mass in kilograms (kg). Based on the WHO BMI criteria, nutritional status was categorized into underweight (BMI < 18.5 kg/m^2^), normal weight (BMI = 18.5–24.9 kg/m^2^), overweight (BMI = 25.0–29.9 kg/m^2^), and obese (BMI ≥ 30.0 kg/m^2^) [39].

### Stool Sample Collection and Processing

Stool samples were self-collected by participants using a standardised procedure. Commercial collection kits and instructions were provided and explained by the trained interviewers in the native language of the participant. We modified the self-collection stool sample instructions used by the National Health Services in the United Kingdom. Specifically, all study participants were provided with clingfilm, disposable gloves, a commercial collection kit, and a clean transparent plastic bag. Participants were instructed to stretch the clingfilm securely over the toilet to collect their stool sample and if possible, to keep the sample free from urine. Next, the participants were instructed to use the spatula inside the lid of the container and transfer a small sample of the stool (approximately the size of two large dates) into the container. The container was then placed in the plastic bag and then handed back to the research assistant. All containers and plastic bags were pre-barcoded with the participants unique study identification number. If participants self-collected samples outside the core working hours of the research team (i.e. late-night or early morning), then they were instructed to leave the plastic bag and sample container in the refrigerator (4 °C) provided so that it could be collected by the research team the following morning. All stool samples were either collected on the same day or the day after and transported for analysis to the Microbiology Laboratory at UAE University. Stool samples were stored at 4 °C and processed within one to 2 days, using a stool concentration technique for ova, cyst, and larvae microscopy detection. As for molecular analyses, two Eppendorf tubes were filled with stool and stored at − 20 °C. The remaining stool samples were kept at 4 °C, for 2 weeks for intestinal coccidian identification, using the modified Ziehl–Neelsen (mZN) stain technique (RAL Diagnostics, France).

### IPs Identification

As previously mentioned, all samples were pre-barcoded with the participants unique study identification number and laboratory staff performed sample preparation and analysis blinded to participant details. Within 1 to 2 days before microscopic examination, all stool samples were examined macroscopically for consistency, colour, and presence of adult worms. Helminths and protozoans were investigated by microscopy and molecular techniques.

#### Microscopic Investigation: Stool Concentration Method

Stool samples were processed following the formol-ether concentration technique for the presence of ova or cysts using the stool Ova/Parasite Concentration Kit (Epitope Diagnostics lnc., FPC200, USA) according to the manufacturer’s instructions. Briefly, approximately 1 g of stool was emulsified with 3% formalin using a vortex. Then 1 ml of ethyl acetate was added and mixed for 10 s by vortex. The stool sample was filtered by a mesh provided with the kit, then centrifuged at 300–350×*g* for 5 min. Sediment was collected and supernatant was decanted. The sediment stool was mixed with three drops of iodine (Logul’s solution, Sigma-Aldrich; Switzerland). The iodine-stained sediment was examined microscopically (Olympus BX53; Germany), under × 10 and × 40 magnification objectives.

#### Modified Ziehl–Neelsen Stain

Cold Ziehl–Neelsen stain (RAL Diagnostics, France) was used for the detection of coccidian species. All slides were examined by an expert microbiologist from the University as well as by a clinical microbiologist from Tawam hospital in Al Ain. *Cryptosporidium* infected slides were used as positive controls.

All tests were repeated three times for quality assurance by two independent microbiologists.

### Molecular Investigation

#### DNA Extraction

The QIAamp DNA Stool Mini Kit (Qiagen, GmbH, Germany) was used for DNA extraction according to manufacturer’s instructions. Briefly, approximately 200 mg of stool was weighed in a two ml microcentrifuge tube. Stool lysing buffer was added to each sample. All samples were heated for 5 min at 70 °C then centrifuged at 14,000 rpm. The supernatant was pipetted into a two ml microcentrifuge tube. An inhibitEX tablet was added to each sample, after which they were centrifuged to pellet inhibitors bound to the InhibitEX matrix. Next, 200 µl of the supernatant was mixed with 15 µl proteinase-K in a 1.5 ml microcentrifuge tube. After this, 200 µl protein precipitating AL buffer was added and vortexed for 15 s. Samples were incubated at 70 °C for 10 min and 200 µl of 100% ethanol was added to the lysate and mixed well. Then, lysate was transferred to a QIAamp spin column and centrifuged for 1 min. Washing buffers AW1 and AW2 were added sequentially to remove salts. Finally, 200 µl of Elution buffer AE was added directly to the QIAamp membrane and incubated for 1 min at room temperature. Extracted DNA was stored at − 20 °C for genome amplification.

### DNA Amplification

Primer-pairs targeting 15 IPs (nine helminths “*A. lumbricoides*, *T. trichiura*, *Necator americanus*, *Strongyloides stercoralis*, *Ancylostoma duodenale*, *Hymenolepis nana*, *Taenia saginata*, *Enterobius vermicularis*, and *Fasciola hepatica*" and six protozoans “*Balantiduim coli, Entamoebea* species*, Giardia lambelia, Cryptospriduim* species*, Isospora* and *C. cayetanensis*”) were used in a polymerase chain reaction (PCR) assay. Primers were obtained from previously published studies, their sequences and expected amplicon sizes are listed in Table 1. DNA extracts were amplified using target-matching PCR assay. Single-plex PCR assays were performed with primer concentrations selected for optimal amplification. Briefly, the amplification reaction mixture consisted of Taq polymerase (Qiagen GmbH, Germany), primers and 2 µl of template DNA in a total volume of 25 µl PCR master mix. DNA amplified using a Bio-Rad T100™ Thermal Cycler for 5 min at 94 °C followed by 40 cycles of 94 °C for 30 s, 56 °C for 30 s and 72 °C for 1 min. Gel electrophoresis on 2% agarose gel was conducted using 1 × TBE electrophoresis buffer (0.1 M Tris, 0.09 M boric acid, 1 mM EDTA). A 50 bp DNA Step Ladder (Promega; Germany) used determine the band size. Sterile water was used as a negative control.

**Table 1:** List of conventional–PCR primer sequences

Target organism	Target gene	Oligonucleotide sequence 5′–3′		Annealing tem. (°C)	Expected bp	Observed bp	Source
*Cryptosporidium* spp.	18S rRNA	F	ATGACGGGTAACGGGGAAT	55	158	170	[54]
		R	CCAATTACAAAACCAAAAAGTCC				
*Entamoeba* spp.	18S rRNA	F	AAACGATGTCAACCAAGGATTG	56	134	140	[53]
		R	TCCCCCTGAAGTCCATAAACTC				
*Taenia saginata*	COX1	F	GGTCATCCAGAGGTTTATG	55	130	140	[55]
		R	CACACTATTGAAAACATAGCAAA				
*Taenia solium*	pTsol9 repetitive element	F	CAGGGTGTGACGTCATGG	55	120	150	[56]
		R	AGGAGGCCAGTTGCCTAGC				
*Trichuris trichuria*	18S	F	TTGAAACGACTTGCTCATCAACTT	58	75	70	[57]
		R	CTGATTCTCCGTTAACCGTTGTC				
*Gardia lamblia*	(16S-like) RNA	F	GACGGCTCAGGACAACGGTT	55	62	70	[57, 58]
		R	TTGCCAGCGGTGTCCG				
*Ascaris lumbricoides*	ITS1	F	GCCACATAGTAAATTGCACACAAAT	56	133	160	[53]
		R	GCCTTTCTAACAAGCCCAACAT				
*Enterobius vermicularis*	5S rRNA gene-IGS region	F	ACAACACTTGCACGTCTCTTC	55	126	130	[59]
		R	TAATTTCTCGTTCCGGCTCA				
*Ancylostoma duodenale*	ITS2	F	GAATGACAGCAAACTCGTTGTTG	57	70	–	[60]
		R	ATACTAGCCACTGCCGAAACGT				
*Necator americanus*	ITS2	F	CTGTTTGTCGAACGGTACTTGC	57	100	–	[60]
		R	ATAACAGCGTGCACATGTTGC				
*Strongyloides stercoralis*	Dispersed repetitive sequence	F	TCCAGAAAAGTCTTCACTCTCCAG	58	85	–	[53]
		R	TGCGTTAGAATTTAGATATTATTGTTGCT				
*Cyclospora cayetanensis*	18S rRNA	F	TAGTAACCGAACGGATCGCATT	55	100	–	[61, 62]
		R	AATGCCACGGTAGGCCAATA				
*Isospora belli*	ITS2	F	ATATTCCCTGCAGCATGTCTGTTT	57	89	–	[63]
		R	CCACACGCGTATTCCAGAGA				
*Fasciola* spp.	ITS2	F	TTGGTACTCAGTTGTCAGTGTG	57	139	–	[64]
		R	AGCATCAGACACATGACCAAG				
*Hymenolepis* spp.	CO1	F	TGGTTTTTTGTGCATCCTGAGGTTTA	42	391	–	[65]
		R	AGAAAGAACGTAATGAAAATGAGCAAC				

### Statistical Analysis

For the descriptive analyses, categorical variables are presented as frequencies and percentages and continuous variables are presented as mean ± standard deviation (SD). We quantified the prevalence of each tested IPs by the type of testing assay (microscopic or molecular), prevalence regardless of the testing assay for being positive for at least one of the tested IPs, and the overall prevalence for being positive for at least one IPs regardless of the testing assay.

The prevalence for being positive for at least one IP regardless of the testing assay, was also quantified according to the workers’ sociodemographic characteristics. Differences in IP prevalence were compared using the Chi square or Fisher’s exact tests. Odds ratio (OR) and adjusted OR were quantified using univariate and multivariate binary logistic regression analyses to assess the crude and adjusted strength of association between being positive for at least one of the tested IPs regardless of the testing assay with workers’ sociodemographic characteristics.

All analyses were conducted using SPSS version 25.0. An alpha value of ≤ 0.05 was considered to indicate statistical significance.

## Results

### Sociodemographic and Health Characteristics

Of the 115 expatriate workers employed in the workplace, 102 expatriate workers consented to participate (88.7% response rate) and 86 provided a stool sample (74.8% response rate). Nearly all participants were males (97.1%), the mean age of the sample was 35.0 years, and the majority (69.3%) were aged between 20 and 39 years. Nearly a third (32.0%) had completed high school or above, 78.4% were from Asian countries (Bangladesh: 37.2% and India: 22.1%). Two thirds (66.7%) of expatriate workers had been working in the UAE for over 5 years and 79.4% were living in communal labour accommodation. On average, six workers shared the same bedroom and 80.2% of the sample shared the same toilet with at least five other people (Table 2).

**Table 2:** Sociodemographic and lifestyle-related characteristics of surveyed workers and prevalence of at least one intestinal parasite by molecular and microscopy assays

Characteristic	N	Valid %	Prevalence (tested = 86)					
			Molecular		Microscopy		Any assay	
			Positive^a^	%	Positive^a^	%	Positive^b^	%
All	102	100	36	41.8	15	17.4	41	40.2
Age (mean = 35.0 years)								
20–29	29	28.7	8	22.2	4	28.6	9	22.5
30–39	41	40.6	18	50.0	6	42.9	19	47.5
40–50	23	22.8	7	19.4	4	28.6	9	22.5
50–56	8	7.9	3	8.3	0	0.0	3	7.5
*P* value				0.905		0.557		0.882
Missing	1		1		1		1	
Education								
Grade 5 or below	20	19.8	11	30.6	5	35.7	12	30.0
Grade 6–8	17	16.8	8	22.2	3	21.4	9	22.5
Grade 9–10	32	31.7	8	22.2	3	21.4	9	22.5
High school or above	32	31.7	9	25.0	3		10	25.0
*P*-value				0.154		0.457		0.115
Missing	1		1		1		1	
Gender								
Male	99	97.1	35	97.2	14	93.3	2	95.1
Female	3	2.9	1	2.8	1	6.7	39	4.9
*P*-value				0.665^c^		0.350^c^		0.224^c^
Marital status								
Ever married	74	72.5	29	80.6	12	80.0	34	28.9
Never married	28	27.5	7	19.4	3	20.0	7	17.1
*P*-value				0.197^c^		0.750^c^		0.069^c^
Ethnicity								
African	13	12.9	7	19.4	1	6.7	7	17.1
Arabic	8	7.8	2	5.6	2	13.3	2	4.9
Asian	80	78.4	27	75.0	12	80.0	32	78.0
*P*-value				0.415		0.162		0.718
Place of residence in home country								
Rural	47	46.1	17	47.2	11	73.3	20	48.8
Urban	55	53.9	19	52.8	4	26.7	21	51.2
*P*-value				0.221^b^		0.090		0.829
Length of stay in UAE								
0–5 years	34	33.3	14	38.9	6	40.0	15	36.6
6–10 years	30	29.4	11	30.6	5	33.3	14	34.1
11–20 years	26	25.5	11	30.6	4	26.7	12	29.3
> 20 years	12	11.8	0	0.0	0	0.0	0	0.0
*P*-value				0.052		0.545		0.024
Accommodation								
Labor accommodation	81	79.4	33	91.7	13	86.7	37	90.2
Live alone	5	4.9	1	2.8	1	6.7	2	4.9
Live with a family	16	15.4	2	5.6	1	6.7	2	4.9
*P*-value				*0.303*		*0.632*		*0.152*
Number of people sharing same room (mean = 5.78)								
0–1	9	9.0	1	2.8	0	0.0	1	2.5
2–3	15	15.0	2	5.6	0	0.0	2	5.0
4–5	13	13.0	5	13.9	4	28.6	6	15.0
≥ 6	63	63.0	28	77.8	10	71.4	31	77.5
*P*-value				0.265		0.107		0.131
Missing	2				1		1	
Number of people sharing same toilet								7.3
≤ 5	20	19.8	3	8.3	1	6.7	3	92.7
> 5	81	80.2	33	91.7	14	93.3	38	0.121^*c*^
*P*-value				0.227^c^		0.380^c^		
Missing	1							
Frequency of eating unwashed food items								
Weekly	5	5.1	4	11.1	1	6.7	4	9.8
Monthly	4	4.1	2	5.6	0	0.0	2	4.9
Yearly	7	7.1	3	8.3	2	13.3	4	9.8
Never	82	83.7	27	75.0	12	80.0	31	75.6
*P*-value				0.074		0.534		0.74
Missing	4							
Most frequent source of food								
Arabia restaurant	6	5.9	2	5.6	2	13.3	2	4.9
Filipino restaurant	5	5.0	3	8.3	0	0.0	3	7.3
Home-based cooking	73	72.3	26	72.2	11	73.3	31	75.6
Indian restaurant	15	14.9	5	13.9	2	13.3	5	12.2
Mixed	2	2.0	0	0.0	0	0.0	0	0.0
*P*-value				0.468		0.369		0.398
Missing	1							
Same cutting board used for vegetables and meat								
No	53	53.0	17	48.6	8	53.3	20	50.0
Rarely	47	47.0	18	51.4	7	46.7	20	50.0
*P*-value				0.427^c^		0.541^c^		0.835
Missing	2		1				1	
Last time traveled abroad of UAE								45.0
Within the last year	44	43.6	14	40.0	9	60.0	18	55.0
Within over a year	57	56.4	21	60.0	6	40.0	22	
*P*-value				0.443^c^		0.109^c^		0.641
Missing	1		1				1	

^a^Positive for at least one of the tested parasites

^b^Positive for at least one of the tested parasites regardless of the testing assay

^c^Fisher’s exact test

According to the Bristol stool score, 43.1% of the workers self-rated their stool type as grade 4 (like a sausage or snake, smooth and soft) while 2.9% self-rated that their stool type was grade 7 (watery, no solid pieces; entirely liquid). The overall mean Hb concentration was 14.63 g/dl and 9.4% of expatriate workers were classified as anaemic. Two-thirds of workers were classified as either overweight (45.5%) or obese (12.0%) (Table 3).

**Table 3:** Health-related characteristics of surveyed workers and prevalence of at least one intestinal parasite by molecular and microscopy assays

Characteristic	N	Valid %	Prevalence (tested = 86)					
			Molecular		Microscopy		Any assay	
			Positive^a^	%	Positive^a^	%	Positive^b^	%
Stool type								
1 (separate hard lumps, like nuts)	0	0	–	–	–	–	–	–
2 (sausage-shaped but lumpy)	8	7.8	5	13.9	3	20.0	5	12.2
3 (like a sausage but with cracks on surface)	22	21.6	7	19.4	2	13.3	8	19.5
4 (like a sausage or snake, smooth and soft)	44	43.1	12	33.3	6	40.0	16	39.0
5 (soft blobs with clear-cut edges)	8	7.8	3	8.3	1	6.7	3	7.3
6 (fluffy pieces with ragged edges, a mushy stool)	17	16.7	7	19.4	3	20.0	7	17.1
7 (watery, no solid pieces. Entirely liquid)	3	2.9	2	5.6	0	0.0	2	4.9
*P*-value						0.492		0.804
Missing								
Craving for sugar								
Daily or weekly	19	18.8	8	22.9	4	26.7	10	25.0
Monthly or rarely	57	56.4	18	51.4	7	46.7	20	50.0
Never	25	24.8	9	25.7	4	26.7	10	25.0
*P*-value						0.712		0.514
Missing	1		1				1	
Anemia (mean Hb = 14.63)								
Not anemic (mean Hb = 14.85)	87	90.6	28	84.8	14	93.3	33	86.8
Anemic (mean Hb = 12.5)	9	9.4	5	15.2	1	6.7	5	13.2
*P*-value				0.173^b^		0.542^b^		0.352
Missing	6		3				3	
Nutritional status (mean BMI = 25.75)								
Underweight (BMI < 18.5)	3	3.0	2	5.6	4	26.7	15	36.6
Normal weight (BMI = 18.5–24.9)	40	39.6	15	41.7	1	6.7	2	4.9
Overweight (BMI = 25.0–29.9)	46	45.5	15	41.7	8	53.3	18	43.9
Obese (BMI ≥ 30)	12	11.9	4	11.1	2	13.3	6	14.6
*P*-value				0.793		0.692		0.593
Missing	1							

^a^Positive for at least one of the tested parasites

^b^Positive for at least one of the tested parasites regardless of the testing assay

^c^Fisher’s exact test

### Microscopic Prevalence of IPs

Of the 102 surveyed workers, only 86 (84.3%) provided stool samples. Microscopically, seven species of IPs were identified in the tested stool samples (four helminths: *T. trichiura*, *Taenia* species, hookworm species and *Hymenolepis* species and three protozoans: *Entamoeba coli*, *Entamoeba hartimani*, *Endolimax nana*, *Cryptosporidium* species, and *Giardia lamblia)*.

Overall, 17.4% of the 86 workers that provided a stool sample tested positive for either protozoa or helminths. *Entamoeba* species (four cases of *Entamoba coli,* one case of *E. hartimani,* and two cases of *E. nana*) were the most common IPs identified in 8.1% of expatriate workers, followed by *Cryptosporidium* species (3.5%). *E. histolytica, S. stercoralis, C. cayetanensis*, *Isospora belli, A. lumbricoides, E. vermicularis*, and *F. hepatica* were zero-prevalence microscopically. Two tested stool samples were positive for *Cryptosporidium* cysts using the Zeihl–Neelsen stain (Table 4).

**Table 4:** Descriptive of tested-positive parasitic infection according to the parasite-ascertainment assay (tested N = 86)

Ascertainment assay	Tested positive parasite species or subspecies n (%)																
	*Cryp*. spp.	*E. histolytica*	*Enta*. spp.	*Taenia* spp.		*T. trichuria*	*G. lamblia*	*Strongyloides stercoralis*	*Cyclospora cayetanensis*	*Isospora belli*	*Ascaris lumbricoides*	*Enterobius vermicularis*	*Fasciola hepatica*	*Hymenolepis nana*	Hook worm		Positive for at least one parasite n (%)
				*T. Saginata*	*T. solium*										*Ancylostoma duodenale*	*Necator americanus*	
Microscopy	3 (3.5)	0 (0.0)	7 (8.1)	1 (1.1)		2 (2.3)	1 (1.1)	0 (0.0)	0 (0.0)	0 (0.0)	0 (0.0)	0 (0.0)	0 (0.0)	1 (1.1)	1 (1.1)		15 (17.4)
Conventional PCR	14 (16.3)	0 (0.0)	9 (10.5)	4 (4.7)	1 (1.1)	2 (2.3)	1 (1.1)	0 (0.0)	0 (0.0)	0 (0.0)	5 (5.8)	12 (14.0)	0 (0.0)	NT	0 (0.0)	0 (0.0)	36 (41.8)
Ziehl–Neelsen	2 (2.3)	NA	NA	NA	NA	NA	NA	NA	NA	NA	NA	NA	NA	NA	NA	NA	2 (2.3)
Positive by at least one assay	16 (18.6)	0 (0.0)	13 (15.1)	4 (4.7)	1 (1.1)	2 (2.3)	1 (1.1)	0 (0.0)	0 (0.0)	**0 (0.0)**	5 (5.8)	12 (14.0)	0 (0.0)	1 (1.1)	1 (1.1)		41 (47.8)^a^

*NT* not tested, *NA* not applicable

^a^Positive for at least one of the tested parasites regardless of the testing assay (this is not the row sum). Two workers were positive for *Cryptosporidium* spp. and three for *Entamoeba* spp. by microscopy but not by conventional PCR

**Fig. 1:**
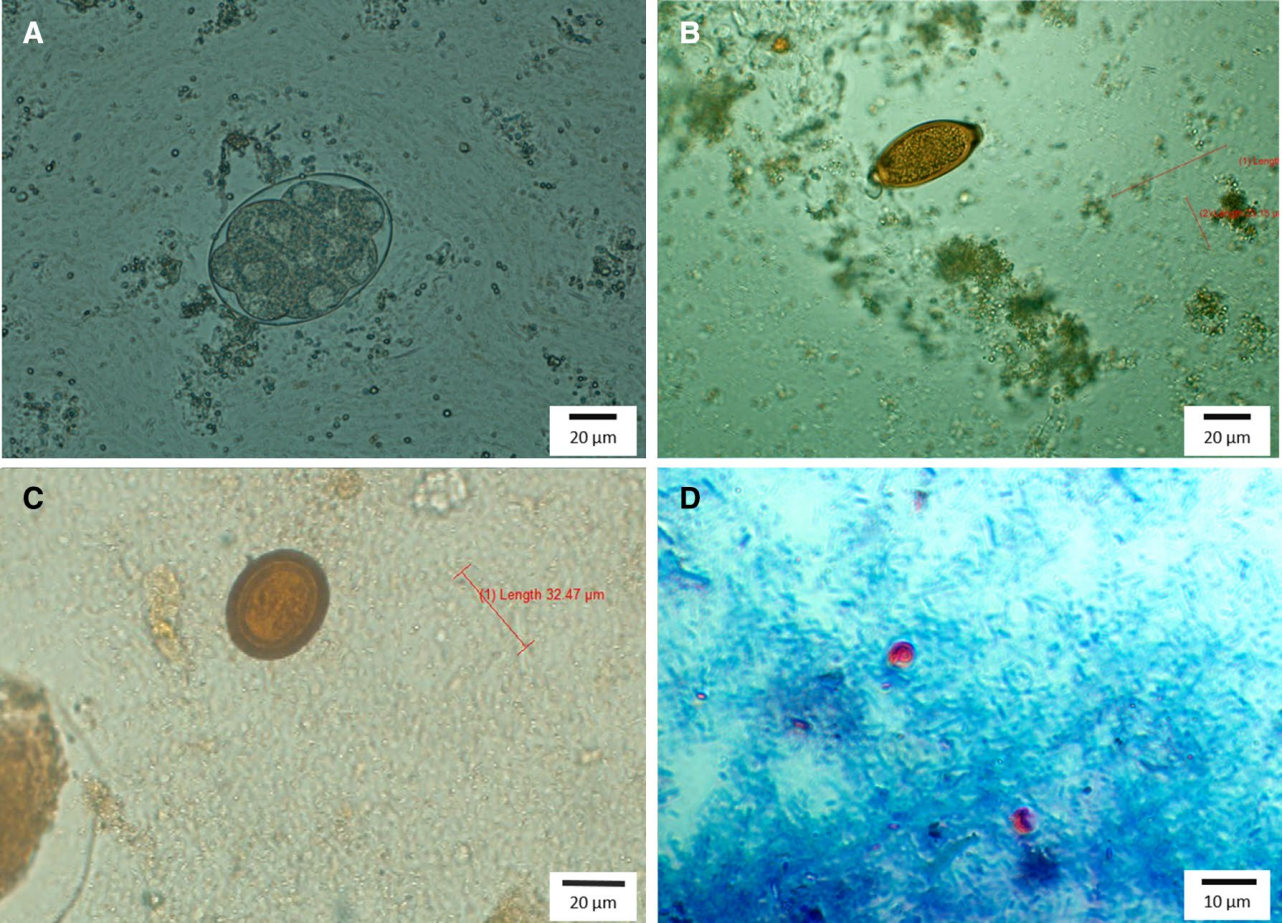
Helminthes eggs and protozoan cysts identified under microscope in tested stool samples. **a***Hookworm*, **b***Trichruis trichiura*, **c***Taenia* species egg identified in a stool using wet mount with iodine. **d***Cryptosporidium* species

Microscopic pictures for some of the identified IPs are shown in Fig. 1.

### Molecular Prevalence of IPs

Fifteen different pathogenic and non-pathogenic IPs species were tested positive by PCR. Nearly half (41.8%) of the tested expatriate workers were positive for at least one of the 15 identified IPs by conventional PCR. The most prevalent parasite was *Cryptosporidium* species (16.3%) followed by *E. vermicularis* (14.0%) and *A. lumbricoides* (5.8%). More than a quarter of expatriate workers were positive for intestinal protozoa (27.9%) and the same proportion tested positive for intestinal helminths (27.9%). Twenty-seven (31.4%) expatriate workers were found to be positive for only one IP, 13 (15.1%) were positive for any two, while only one (1.2%) expatriate worker was positive for any three of the 15 identified IPs.

Regardless of the IPs ascertainment assay, 47.8% of the expatriate workers were positive for at least one of the 15 identified IPs; 34.9% were positive for intestinal protozoa, and 12.8% were positive for intestinal helminths. Among these, 65.8% and 34.1% of expatriate workers had mono- and poly-parasitism, respectively. The most common IPs among the 41 expatriate workers with positive tests were *Cryptosporidium* species (39.0%), followed by *Entamoeba* species (31.7%), and *Entrobious vermicularis* (29.3%). Overall, of the 41 tested positive workers, 73.2% were positive for protozoa and 60.9% were positive for helminths (Table 4).

**Fig. 2:**
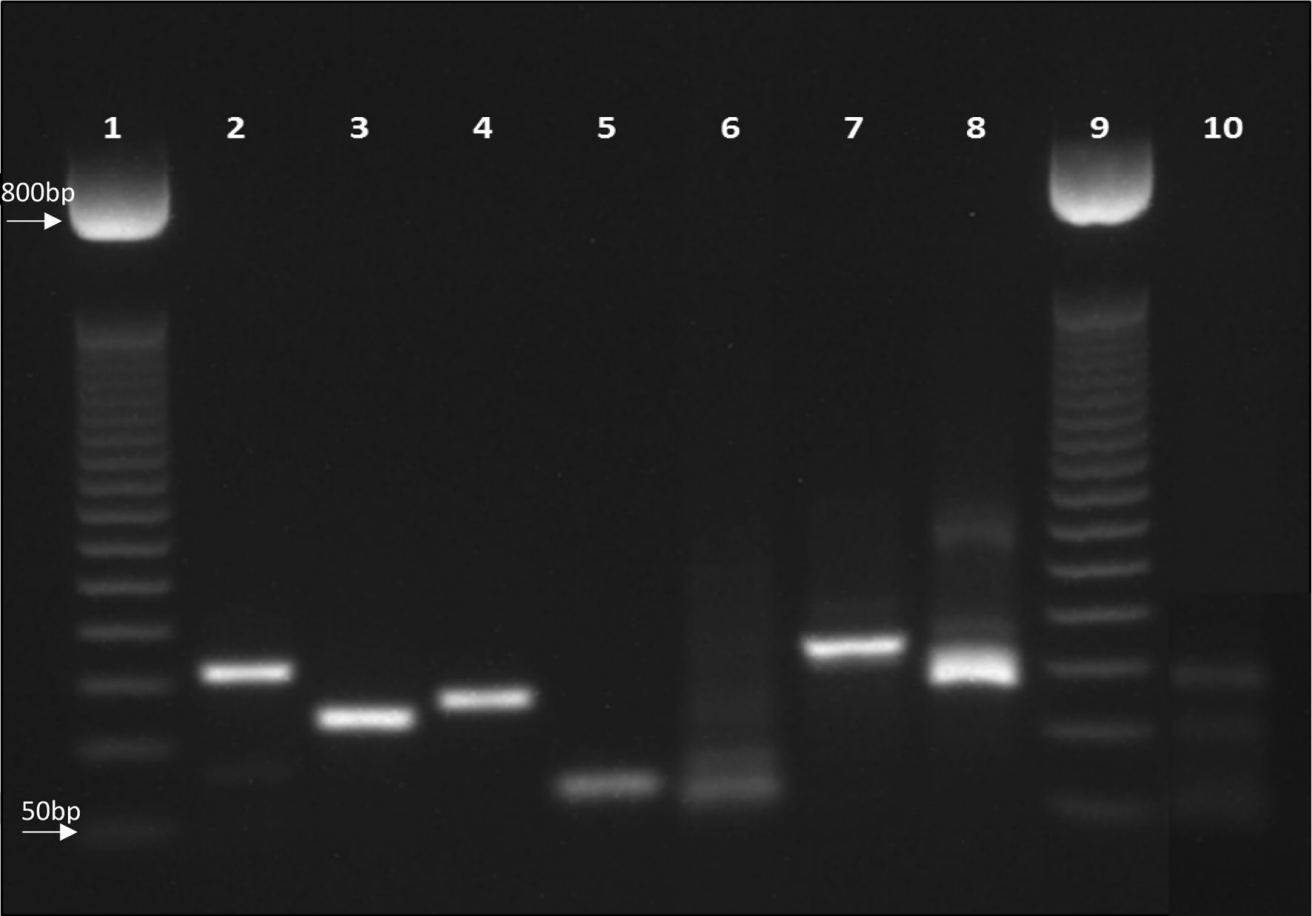
Agarose gel electrophoresis (2%) shows PCR products of intestinal parasites. Lanes 1 and 9: 50 basepair (pb) DNA ladder, lane 2: *A. lumbricoides*, lane 3: *E. vermicularis*, lane 4: *T. saginata*, lane 5: *Gardia lamblia*, lane 6: *Trichuris trichuria*, lane 7: *Cryptosporidium* spp. lane 8: *Taenia solium*, lane 10: *Entamoeba* spp.

PCR product for some of the identified IPs is shown in Fig. 2.

### Factors Associated with IPs

The only factor that was significantly associated with being positive for any of the identified 15 IPs using PCR was educational attainment. Workers that reported an educational attainment of grades 9–10 were 74% (OR 0.26, 95% CI 0.07–0.89) less likely to be positive for any of the identified IPs compared to workers with grade 5 or below. As for the microscopic assay, none of the measured characteristics reached significance. Workers sharing the same toilet with > 5 other workers were more likely to be positive for at least one IPs using microscopic (OR 2.15, 95% CI 0.53–8.74) or molecular assay (OR 2.33, 95% CI 0.28–19.76). Anaemic workers were 168% more likely (OR 2.68%, 95% CI 0.59–12.01) while 40% less likely (OR 0.60, 95% CI 0.07–5.30) to be positive for any of the identified IPs, based on microscopic and molecular assays, respectively. Underweight workers were also more likely to be positive for any of the identified IPs based on microscopic (OR 2.40, 95% CI 0.20–29.13) and molecular assays (OR 3.63, 95% CI 0.26–49.7). These results were consistent when we explored whether being positive for at least one of the identified IPs regardless of the testing assay. Nevertheless, none of these findings reached a significance level at *P* ≤ 0.05 (Table 5).

**Table 5:** Characteristics associated with being positive for at least one intestinal parasite ascertained by conventional PCR and microscopy

Characteristic	Molecular	Microscopy	Any assay
	OR (95% CI)	OR (95% CI)	
Age (mean = 34.96 years)			
20–29	1.00	1.00	1.00
30–39	1.50 (0.51–4.39)	0.82 (0.20–3.28)	1.37 (0.48–3.95)
40–50	1.23 (0.33–4.50)	1.39 (0.29–6.58)	1.63 (0.45–5.82)
50–56	1.31 (0.23–7.41)	–	1.083 (0.19–6.06)
Education			
Grade 5 or below	1.00	1.00	1.00
Grade 6–8	0.64 (0.16–2.48)	0.60 (0.11–3.02)	0.64 (0.16–2.56)
Grade 9–10	0.26 (0.07–0.89)*	0.31 (0.06–1.52)	0.28 (0.07–0.84)
High school or above	0.41 (0.11–1.45)	0.39 (0.08–192)	0.39 (0.11–1.38)
Gender			
Male	1.00	1.00	1.00
Female	1.40 (0.08–23.15)	5.00 (0.29–84.76)	–
Marital status			
Ever married	1.00	1.00	1.00
Never married	0.56 (0.20–1.57)	0.68 (0.17–2.69)	0.41 (0.15–1.15)
Ethnicity			
African	1.00	1.00	1.00
Arabic	0.71 (0.07–6.92)	11.00 (0.65–187.16)	0.71 (0.07–6.92)
Asian	0.45 (0.13–1.56)	2.27 (0.27–19.33)	0.60 (0.17–2.08)
Place or residence in home country			
Rural	1.00	1.00	1.00
Urban	1.54 (0.66–3.67)	0.35 (0.10–1.22)	1.10 (0.47–2.56)
Length of time in UAE			
0–5 years	1.00	1.00	1.00
6–10 years	0.84 (0.29–2.41)	0.95 (0.25–3.58)	1.17 (0.41–3.34)
11–20 years	1.26 (0.41–3.84)	0.94 (0.23–3.85)	1.33 (0.43–4.10)
> 20 years	NA	NA	NA
Accommodation			
Labor accommodation	1.00	1.00	1.00
Live alone	0.61 (0.05–6.98)	2.31 (0.19–27.39)	1.95 (0.17–22.41)
Live with a family	0.30 (0.06–1.53)	0.51 (0.06–4.41)	0.24 (0.05–1.22)
Number of people sharing same room (mean = 5.78)			
≥ 6	1.00	1.00	1.00
4–5	0.89 (0.25–3.26)	2.74 (0.67–11.17)	1.05 (0.29–3.81)
2–3	0.31 (0.06–1.60)	NA	0.25 (0.5–1.30)
0–1	0.21 (0.02–1.95)	NA	0.17 (0.02–1.56)
Number of people sharing same toilet			
≤ 5	1.00	1.00	1.00
> 5	2.15 (0.53–8.74)	2.33 (0.28–19.76)	2.81 (0.69–11.45)
Frequency of eating unwashed food items			
Weekly	1.00	1.00	1.00
Monthly	NA	NA	NA
Yearly	0.38 (0.02–6.35)	2.67 (0.16–45.11)	1.00 (0.05–22.17)
Never	0.15 (0.02–1.41)	0.81 (0.08–7.80)	0.19 (0.02–1.77)
Most frequent source eaten food			
Arabia restaurant	1.00	1.00	1.00
Filipino restaurant	3.0 (0.15–59.98)	NA	3.00 (0.15–59.89)
Home-based cooking	0.74 (0.09–5.62)	0.22 (0.02–1.73)	1.03 (0.13–7.82)
Indian restaurant	0.56 (0.06–5.24)	0.16 (0.01–1.96)	0.55 (0.06–5.24)
Mixed	NA	NA	NA
Use same cutting board for vegetables and meat			
No	1.00	1.00	1.00
Rarely	1.20 (0.50–2.85)	0.90 (0.29–2.76)	1.10 (0.46–2.58)
Last time traveled abroad of UAE			
Within the last year	1.00	1.00	1.00
Within over a year	1.18 (0.49–2.83)	0.42 (0.13–1.31)	0.82 (0.34–1.93)
Stool type			
2 (sausage-shaped but lumpy)	1.00	1.00	1.00
3 (like a sausage but with cracks on surface)	0.26 (0.04–1.69)	0.17 (0.02–1.36)	0.32 (0.05–2.11)
4 (like a sausage or snake, smooth and soft)	0.20 (0.03–1.19)	0.37 (0.05–1.51)	0.32 (0.05–1.87)
5 (soft blobs with clear-cut edges)	0.30 (0.03–2.76)	0.22 (0.02–2.97)	0.30 (0.03–2.75)
6 (fluffy pieces with ragged edges, a mushy stool)	0.35 (0.05–2.41)	0.33 (0.05–2.37)	0.35 (0.05–2.41)
7 (watery, no solid pieces. Entirely liquid)	0.80 (0.04–14.6)		0.80 (0.04–14.6)
Craving for sugar			
Daily or weekly	1.00	1.00	1.00
Monthly or rarely	0.70 (0.23–2.14)	0.57 (0.14–2.26)	0.52 (0.17–1.60)
Never	0.84 (0.23–3.05)	0.76 (0.16–3.65)	0.64 (0.18–2.31)
Anemia (mean Hb = 14.63)			
Not anemic (mean Hb = 14.85)	1.00	1.00	1.00
Anemic (mean Hb = 12.5)	2.68 (0.59–12.01)	0.60 (0.07–5.30)	2.02 (0.45–9.09)
Nutritional status (mean BMI = 25.75)			
Normal weight (BMI = 18.5–24.9)	1.00	1.00	1.00
Underweight (BMI < 18.5)	2.40 (0.20–29.13)	3.63 (0.26–49.7)	2.40 (0.19–29.1)
Overweight (BMI = 25.0–29.9)	0.72 (0.28–1.84)	1.81 (0.49–6.65)	0.98 (0.39–2.48)
Obese (BMI ≥ 30)	0.96 (0.22–4.22)	2.07 (0.31–13.67)	2.40 (0.51–11.3)

*OR* crude odds ratio, *NA* not applicable due to the zero prevalence

*P < 0.05

## Discussion

Findings from this study revealed a high prevalence of IPs in expatriate workers in an industrial district in Al Ain city, UAE. Almost half (47.8%) of workers were positive for at least one of the identified 15 tested intestinal helminths and protozoal parasites. The prevalence of the tested IPs was higher among workers with an educational attainment of grade 5 or below, from Asian countries, were living in rural settings in their home country, currently living in labour accommodation, sharing the same bedroom with ≥ 6 other workers, or sharing the same toilet with > 5 other people.

The microscopically observed overall prevalence (17.4%) of at least one of the identified IPs in the surveyed expatriate workers is 5.3-times higher than that observed in expatriate workers in Sharjah Emirate (UAE) in 2008 [40] and 2013 [36]. This prevalence is slightly higher than that reported in expatriates working in Saudi Arabia (14.9%) in 2013 [41]. In other studies in the UAE [40], Saudi Arabia [41], and Qatar [42], the IPs prevalence dropped to less than 10%, in long-term residents and expatriates. It is important to note there are foci in Saudi Arabia [27], Oman [43], and Yemen [44] that have a higher incidence of IPs naturally when compared to other parts of these countries. This may be due to geographical locations with tropical climates or rural, isolated areas.

*Cryptosporidium* infection cases were highest when detected by PCR (14 cases; 16.2%). However, only two samples were observed under the microscope most probably due to the low number of *Cryptosporidium* count in the stool. *Cryptosporidium* is the most common water-borne IP in the world and it causes severe diarrhoea [16]. However, the *Cryptosporidium* genus consists of at least 16 species, with two species, *C. hominis* and *C. parvum*, causing most cases of cryptosporidiosis in humans [45]. *Cryptosporidium* must have 50,000–500,000 oocysts in formed or semi-formed stool to be detected under the microscope [46]. Other *Cryptosporidium* species have been shown to cause illness in humans, including *Cryptosporidium meleagridis*, *Cryptosporidium felis* and *Cryptosporidium canis* [47], however, in our study we were only able to investigate the *Cryptosporidium* species and not the exact genus.

IPs have to reach a threshold to be visualised by microscopy and cause clinical or sub-clinical symptoms. Anaemic workers were 168% more likely to be positive for any of the detected IPs, based on only microscopic detection. Other IP-positive workers could be less likely to be classified as anaemic using pulse oximetry if they are smokers [48]. Pinworm is the second most common infection in human [49]. In our study, *E. vermicularis* was only detected by PCR in 12 cases; however, it was not found by microscopy or macroscopy. Most of *E. vermicularis* infections are asymptomatic and are commonly seen amongst school children. Microscopically, *E. vermiculars* eggs are rarely detected in stool because gravid females deposit eggs in a sticky film directly onto the perianal skin at night [50].

Entamoeba genus consist of many species, six of which (*E. histolytica*, *E. dispar*, *E. moshkoviskii*, *E. coli*, *E. hartmani*, and *E. polki*) reside in the human intestinal lumen. *E. histolytica* is the only pathogenic species that causes intestinal and extra intestinal amebiasis, this is a common parasitic cause of significant morbidity and mortality in developing countries which is usually transmitted from person to person through faecal–oral contaminated food or hands [51, 52]. In our study, all of the screened stool samples were negative for the *E. histolytica* using PCR. However, using more common primers that can amplify genomic materials of *E. dispar*, *E. bangladishi*, *E. histolytica* and *E. moshkovskii* [53], nine stool samples were positive. This higher than expected prevalence (10.5%) for non-pathogenic *Entamoeba* species is probably an indication of the poor personal hygiene habits and lifestyle among this sample of expatriate workers. Indeed, poor personal hygiene habits could potentially expose workers to more serious IPs and illnesses.

Except for the negative association between educational attainment and molecular-ascertainment for at least one of the identified IPs, none of the measured sociodemographic or health-related characteristics revealed significant associations regardless of the IP-ascertainment assay. This finding could be attributed to two potential reasons. Firstly, the study was underpowered to detect associations between these characteristics and IPs. Secondly, this might indicate that workers of different socio-demographic and health-related characteristics have an equal opportunity of exposure to IPs. This assumption is supported by the fact that all of the tested expatriate workers were selected from the same working place in the same industrial district where most of them (79.4%) lived in labor accommodation, 91.0% shared bed rooms with at least another two workers, and 80.2% of them shared toilets with > 5 other workers. Our study suggests potential clustering of intestinal parasitic infections amongst expatriate workers sharing the same bedroom and/or bathroom and future studies would do well to specifically explore the potential clustering of cases.

The present survey improved the reliability and validity of the results through the use of well-trained, native-speaking interviewers, standardized questionnaires, confidential anonymous interviews, and maintaining good communication between the research team and employers throughout the research process. The reliability and validity of identifying workers infected with or carrying IPs was improved through testing stool samples via well-trained and expert microbiologists using both microscopy and molecular assays. Moreover, the molecular assays used helped to quantify the burden of IPs not only in intensively infected workers, but also in workers who carried a low load of IPs that would not be able to be detected microscopically.

The present findings should be interpreted in view of several potential limitations. The cross-sectional design limits the observed association pathway between being positive for at least one IPs and education level. Testing expatriate workers from only one industrial area limits the generalizability of the findings to other workers in different emirates and even to the general population. Despite these limitations, the current findings advocate public health awareness and intervention campaigns to control IPs and promote public health, especially in migrant workers with low education levels originating from countries with a high prevalence of IPs.

## Conclusion

Our sample of expatriate workers in Al Ain industrial district were burdened with a high prevalence of IPs. Concerted efforts to control IPs at the industrial district-level would help to protect multinational workers’ health and well-being. Moreover, public health programs targeting reducing IP prevalence have the potential to reduce unfavorable health consequences, increase worker’s productivity, and avoid the spread of IPs to the general public. Educational programs could be directed towards improving workers’ health education and in particular the importance of practicing a hygienic lifestyle. This study advocates for large scale studies to estimate the burden of IPs in the general UAE population at national and subnational levels.
